# Bis[(*E*)-1-(3,4-dichloro­benzyl­idene­amino)-4-methyl­pyridinium] bis­(maleonitrile­dithiol­ato)nickelate(II)

**DOI:** 10.1107/S1600536809001159

**Published:** 2009-01-17

**Authors:** Jian-Lan Liu, Bing-Qian Yao, Qi Liu, Shao-Ming Zhang

**Affiliations:** a Department of Applied Chemistry, College of Sciences, Nanjing University of Technology, Nanjing 210009, People’s Republic of China

## Abstract

The asymmetric unit of the title compound, (C_13_H_11_Cl_2_N_2_)_2_[Ni(C_4_N_2_S_2_)_2_], contains one-half of a centrosymmetric [Ni(mnt)_2_] anion (where mnt is maleonitrile­dithiol­ate or 1,2-dicyano-1,2-ethyl­enedithiol­ate) and an (*E*)-1-(3,4-dichloro­benzyl­ideneamino)-4-methyl­pyridinium cation. In the anion, the coordination around the Ni atom is a distorted square. In the cation, the aromatic rings are oriented at a dihedral angle of 7.81 (3)°. In the crystal structure, inter­molecular C—H⋯N hydrogen bonds link the cations and anions. π–π Contacts between the nickel dithiol­ene and pyridine rings and between the benzene and pyridine rings, [centroid–centroid distances = 3.682 (3) and 3.643 (3) Å, respectively] may further stabilize the structure.

## Related literature

For general background, see: Robertson & Cronin (2002 ▶); Cassoux *et al.* (1991 ▶). For bond-length data, see: Allen *et al.* (1987 ▶).
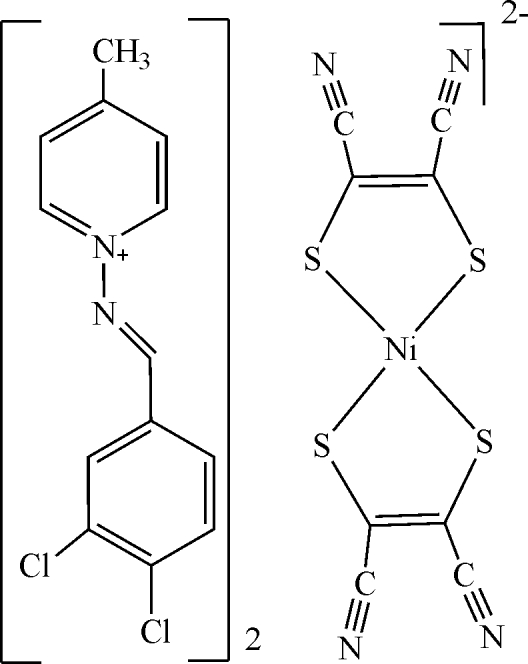


## Experimental

### 

#### Crystal data

(C_13_H_11_Cl_2_N_2_)_2_[Ni(C_4_N_2_S_2_)_2_]
*M* *_r_* = 871.37
Monoclinic, 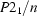

*a* = 10.7054 (10) Å
*b* = 13.8664 (13) Å
*c* = 12.5043 (12) Å
β = 95.803 (1)°
*V* = 1846.7 (3) Å^3^
*Z* = 2
Mo *K*α radiation
μ = 1.08 mm^−1^
*T* = 296 (2) K
0.30 × 0.20 × 0.10 mm

#### Data collection

Bruker SMART CCD area-detector diffractometer
Absorption correction: multi-scan (*SADABS*; Bruker, 2000 ▶) *T*_min_ = 0.729, *T*_max_ = 0.895
15877 measured reflections
4273 independent reflections
3611 reflections with *I* > 2σ(*I*)
*R*_int_ = 0.023

#### Refinement

*R*[*F* ^2^ > 2σ(*F* ^2^)] = 0.033
*wR*(*F* ^2^) = 0.089
*S* = 1.08
4273 reflections
232 parameters
H-atom parameters constrained
Δρ_max_ = 0.44 e Å^−3^
Δρ_min_ = −0.37 e Å^−3^

### 

Data collection: *SMART* (Bruker, 2000 ▶); cell refinement: *SAINT* (Bruker, 2000 ▶); data reduction: *SAINT*; program(s) used to solve structure: *SHELXS97* (Sheldrick, 2008 ▶); program(s) used to refine structure: *SHELXL97* (Sheldrick, 2008 ▶); molecular graphics: *SHELXTL* (Sheldrick, 2008 ▶); software used to prepare material for publication: *SHELXTL*.

## Supplementary Material

Crystal structure: contains datablocks global, I. DOI: 10.1107/S1600536809001159/hk2608sup1.cif

Structure factors: contains datablocks I. DOI: 10.1107/S1600536809001159/hk2608Isup2.hkl

Additional supplementary materials:  crystallographic information; 3D view; checkCIF report

## Figures and Tables

**Table:** 

Ni1—S1	2.1622 (5)
Ni1—S2	2.1838 (5)

**Table:** 

S1—Ni1—S2^i^	88.128 (19)
S1—Ni1—S2	91.872 (19)

Symmetry code: (i) 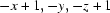.

**Table 2:** Hydrogen-bond geometry (Å, °)

*D*—H⋯*A*	*D*—H	H⋯*A*	*D*⋯*A*	*D*—H⋯*A*
C5—H5*A*⋯N2^ii^	0.93	2.51	3.413 (3)	163

Symmetry code: (ii) 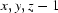.

**Fig. 1.**
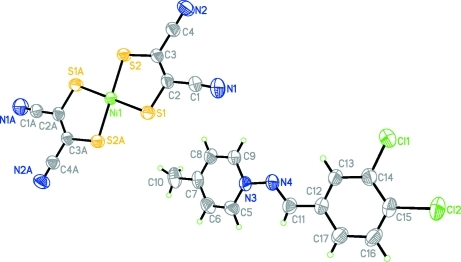
The molecular structure of the title molecule, with the atom-numbering scheme. Displacement ellipsoids are drawn at the 50% probability level [symmetry code (A): 1 - x, -y, 1 - z].

**Fig. 2.**
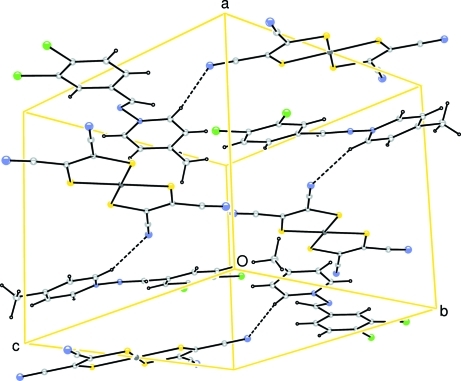
A partial packing diagram of the title compound. Hydrogen bonds are shown as dashed lines.

## supplementary crystallographic information

### Comment

Square-planar *M*[dithiolene]_2_ complexes have attracted extensive interests in the areas of conducting and magnetic materials, dyes, non-linear optics and catalysis (Robertson *et al.*, 2002; Cassoux *et al.*, 1991). We report herein the crystal structure of the title compound.

The asymmetric unit of the title compound (Fig. 1) contains one-half of centrosymmetric [Ni(mnt)_2_] (where mnt is maleonitriledithiolate) anion and a (*E*)-1-(3,4-di-chlorobenzylideneamino)-4-methylpyridinium cation. In the anion, the coordination around the Ni atom is a distorted square (Table 1). The bond lengths (Allen *et al.*,  1987) and angles are within normal ranges. Rings A (Ni1/S1/S2/C2/C3), B (N3/C5-C9) and C (C12-C17) are, of course, planar and they are oriented at dihedral angles of A/B = 16.69 (3)°, A/C = 13.47 (3)° and B/C = 7.81 (3)°.

In the crystal structure, intermolecular C-H···N hydrogen bonds (Table 2) link the cations and anions (Fig. 2), in which they may be effective in the stabilization of the structure. The π-π contacts between the nickel dithiolene and the pyridine rings and the benzene and the pyridine rings, Cg1—Cg3^i^ and Cg3—Cg4^ii^ [symmetry codes: (i) 1/2 + x, 1/2 - y, 1/2 + z; (ii) 1 - x, 1 - y, -z, where Cg1, Cg3 and Cg4 are centroids of the rings A (Ni1/S1/S2/C2/C3), B (N3/C5-C9) and C (C12-C17), respectively] may further stabilize the structure, with centroid-centroid distances of 3.682 (3) Å and 3.643 (3) Å.

### Experimental

For the preparation of the title compound, disodium maleonitriledithiolate (458 mg, 2.46 mmol) and nickel chloride hexahydrate (230 mg, 0.96 mmol) were mixed by stirring in EtOH (20 ml) at room temperature. Subsequently, a solution of (*E*)-1-(3,4-di-chlorobenzylideneamino)-4-methylpyridinium iodide (2143 mg, 2.46 mmol) in EtOH (10 ml) was added to the mixture, and the red precipitate immediately formed was filtered off, and washed with EtOH. The crude product was recrystallized in acetone (20 ml) to give black crystals. Crystals suitable for X-ray analysis were obtained by diffusing diethyl ether into the solution of the title compound in acetone for 8 d. FT-IR data (KBr pellets, cm^-1^): 2189 (*s*), 2920 (*s*),1631(*s*), 1485 (*s*), 1272 (*s*).

### Refinement

H atoms were positioned geometrically, with C-H = 0.93 and 0.96 Å for aromatic and methyl H, respectively, and constrained to ride on their parent atoms, with U_iso_(H) = xU_eq_(C), where x = 1.5 for methyl H and x = 1.2 for aromatic H atoms.

### Crystal data

**Table:**

(C_13_H_11_Cl_2_N_2_)_2_[Ni(C_4_N_2_S_2_)_2_]	*F*(000) = 884
*M**_r_* = 871.37	*D*_x_ = 1.567 Mg m^−^^3^
Monoclinic, *P*2_1_/*n*	Mo *K*α radiation, λ = 0.71073 Å
Hall symbol: -P 2yn	Cell parameters from 3049 reflections
*a* = 10.7054 (10) Å	θ = 2.1–22.4°
*b* = 13.8664 (13) Å	µ = 1.08 mm^−^^1^
*c* = 12.5043 (12) Å	*T* = 296 K
β = 95.803 (1)°	Block, black
*V* = 1846.7 (3) Å^3^	0.30 × 0.20 × 0.10 mm
*Z* = 2	

### Data collection

**Table:**

Bruker SMART CCD area-detector diffractometer	4273 independent reflections
Radiation source: fine-focus sealed tube	3611 reflections with *I* > 2σ(*I*)
graphite	*R*_int_ = 0.023
φ and ω scans	θ_max_ = 27.6°, θ_min_ = 2.2°
Absorption correction: multi-scan (*SADABS*; Bruker, 2000)	*h* = −13→13
*T*_min_ = 0.729, *T*_max_ = 0.895	*k* = −18→18
15877 measured reflections	*l* = −13→16

### Refinement

**Table:**

Refinement on *F*^2^	Primary atom site location: structure-invariant direct methods
Least-squares matrix: full	Secondary atom site location: difference Fourier map
*R*[*F*^2^ > 2σ(*F*^2^)] = 0.033	Hydrogen site location: inferred from neighbouring sites
*wR*(*F*^2^) = 0.089	H-atom parameters constrained
*S* = 1.08	*w* = 1/[σ^2^(*F*_o_^2^) + (0.044*P*)^2^ + 0.5603*P*] where *P* = (*F*_o_^2^ + 2*F*_c_^2^)/3
4273 reflections	(Δ/σ)_max_ = 0.001
232 parameters	Δρ_max_ = 0.44 e Å^−^^3^
0 restraints	Δρ_min_ = −0.37 e Å^−^^3^

### Special details

**Table:**

Geometry. All e.s.d.'s (except the e.s.d. in the dihedral angle between two l.s. planes) are estimated using the full covariance matrix. The cell e.s.d.'s are taken into account individually in the estimation of e.s.d.'s in distances, angles and torsion angles; correlations between e.s.d.'s in cell parameters are only used when they are defined by crystal symmetry. An approximate (isotropic) treatment of cell e.s.d.'s is used for estimating e.s.d.'s involving l.s. planes.
Refinement. Refinement of *F*^2^ against ALL reflections. The weighted *R*-factor *wR* and goodness of fit *S* are based on *F*^2^, conventional *R*-factors *R* are based on *F*, with *F* set to zero for negative *F*^2^. The threshold expression of *F*^2^ > σ(*F*^2^) is used only for calculating *R*-factors(gt) *etc*. and is not relevant to the choice of reflections for refinement. *R*-factors based on *F*^2^ are statistically about twice as large as those based on *F*, and *R*- factors based on ALL data will be even larger.

### Fractional atomic coordinates and isotropic or equivalent isotropic displacement parameters (Å^2^)

**Table:**

	*x*	*y*	*z*	*U*_iso_*/*U*_eq_	
Ni1	0.5000	0.0000	0.5000	0.03598 (10)	
Cl1	0.39412 (8)	0.83968 (4)	0.09607 (5)	0.0739 (2)	
Cl2	0.39194 (7)	0.91419 (4)	−0.14262 (5)	0.07117 (18)	
S1	0.48602 (6)	0.12888 (4)	0.40218 (4)	0.05280 (15)	
S2	0.48684 (5)	0.08311 (3)	0.64679 (4)	0.04583 (13)	
N1	0.4615 (3)	0.38988 (15)	0.41030 (19)	0.0831 (7)	
N2	0.4392 (2)	0.33492 (14)	0.73857 (16)	0.0663 (5)	
N3	0.32151 (14)	0.36297 (10)	0.05354 (12)	0.0393 (3)	
N4	0.34563 (15)	0.46221 (11)	0.03826 (13)	0.0449 (4)	
C1	0.4621 (2)	0.31630 (15)	0.45077 (17)	0.0549 (5)	
C2	0.46864 (18)	0.22060 (13)	0.49411 (16)	0.0446 (4)	
C3	0.46740 (17)	0.20093 (13)	0.60048 (15)	0.0407 (4)	
C4	0.45178 (19)	0.27570 (14)	0.67685 (16)	0.0477 (4)	
C5	0.3192 (2)	0.29411 (15)	−0.02278 (18)	0.0543 (5)	
H5A	0.3343	0.3101	−0.0925	0.065*	
C6	0.2946 (2)	0.20068 (15)	0.0028 (2)	0.0584 (5)	
H6A	0.2919	0.1537	−0.0505	0.070*	
C7	0.27374 (19)	0.17458 (14)	0.10617 (19)	0.0517 (5)	
C8	0.2804 (2)	0.24667 (15)	0.18250 (18)	0.0543 (5)	
H8A	0.2682	0.2316	0.2532	0.065*	
C9	0.3045 (2)	0.34025 (14)	0.15582 (16)	0.0487 (4)	
H9A	0.3092	0.3880	0.2083	0.058*	
C10	0.2459 (3)	0.07236 (16)	0.1355 (3)	0.0768 (8)	
H10A	0.2449	0.0323	0.0728	0.115*	
H10B	0.1656	0.0695	0.1631	0.115*	
H10C	0.3096	0.0499	0.1893	0.115*	
C11	0.32672 (19)	0.49507 (13)	−0.05590 (17)	0.0471 (4)	
H11A	0.3002	0.4544	−0.1129	0.056*	
C12	0.34690 (17)	0.59789 (13)	−0.07481 (16)	0.0428 (4)	
C13	0.35970 (18)	0.66292 (14)	0.00916 (16)	0.0457 (4)	
H13A	0.3585	0.6412	0.0794	0.055*	
C14	0.37433 (18)	0.76020 (14)	−0.01073 (16)	0.0461 (4)	
C15	0.37612 (18)	0.79287 (14)	−0.11577 (17)	0.0473 (4)	
C16	0.3660 (2)	0.72771 (16)	−0.19945 (17)	0.0573 (5)	
H16A	0.3692	0.7493	−0.2695	0.069*	
C17	0.3511 (2)	0.63062 (16)	−0.18005 (17)	0.0553 (5)	
H17A	0.3440	0.5871	−0.2369	0.066*	

### Atomic displacement parameters (Å^2^)

**Table:**

	*U*^11^	*U*^22^	*U*^33^	*U*^12^	*U*^13^	*U*^23^
Ni1	0.04103 (18)	0.02949 (16)	0.03748 (18)	−0.00120 (12)	0.00426 (12)	0.00094 (12)
Cl1	0.1235 (6)	0.0405 (3)	0.0601 (3)	0.0083 (3)	0.0212 (3)	−0.0033 (2)
Cl2	0.0980 (5)	0.0409 (3)	0.0771 (4)	−0.0025 (3)	0.0212 (3)	0.0183 (3)
S1	0.0855 (4)	0.0343 (2)	0.0391 (3)	0.0037 (2)	0.0086 (2)	0.00337 (18)
S2	0.0636 (3)	0.0350 (2)	0.0393 (2)	−0.0017 (2)	0.0074 (2)	−0.00029 (18)
N1	0.129 (2)	0.0424 (11)	0.0774 (15)	0.0156 (12)	0.0078 (14)	0.0102 (10)
N2	0.0909 (15)	0.0513 (11)	0.0586 (11)	0.0040 (10)	0.0163 (10)	−0.0105 (9)
N3	0.0427 (8)	0.0299 (7)	0.0456 (8)	0.0002 (6)	0.0058 (6)	0.0016 (6)
N4	0.0546 (9)	0.0300 (7)	0.0506 (9)	−0.0035 (6)	0.0072 (7)	0.0025 (6)
C1	0.0731 (14)	0.0395 (10)	0.0513 (12)	0.0092 (9)	0.0023 (10)	−0.0002 (9)
C2	0.0484 (10)	0.0343 (9)	0.0507 (11)	0.0029 (7)	0.0038 (8)	0.0006 (8)
C3	0.0410 (9)	0.0337 (8)	0.0474 (10)	0.0007 (7)	0.0037 (7)	−0.0032 (7)
C4	0.0551 (11)	0.0401 (10)	0.0486 (11)	0.0005 (8)	0.0083 (9)	−0.0012 (8)
C5	0.0720 (14)	0.0424 (10)	0.0497 (11)	−0.0014 (9)	0.0127 (10)	−0.0057 (9)
C6	0.0685 (14)	0.0383 (10)	0.0690 (14)	−0.0025 (9)	0.0088 (11)	−0.0111 (10)
C7	0.0439 (10)	0.0328 (9)	0.0779 (15)	0.0005 (7)	0.0040 (10)	0.0047 (9)
C8	0.0662 (13)	0.0413 (10)	0.0564 (12)	0.0021 (9)	0.0112 (10)	0.0100 (9)
C9	0.0619 (12)	0.0366 (9)	0.0478 (11)	0.0017 (8)	0.0063 (9)	0.0018 (8)
C10	0.0816 (17)	0.0339 (11)	0.115 (2)	−0.0054 (10)	0.0105 (16)	0.0099 (12)
C11	0.0536 (11)	0.0383 (10)	0.0484 (11)	−0.0039 (8)	0.0008 (8)	0.0031 (8)
C12	0.0422 (9)	0.0382 (9)	0.0475 (10)	−0.0019 (7)	0.0020 (8)	0.0079 (8)
C13	0.0533 (11)	0.0405 (10)	0.0443 (10)	0.0052 (8)	0.0106 (8)	0.0083 (8)
C14	0.0498 (11)	0.0395 (9)	0.0500 (11)	0.0041 (8)	0.0104 (8)	0.0021 (8)
C15	0.0481 (10)	0.0377 (9)	0.0567 (11)	−0.0013 (8)	0.0075 (9)	0.0123 (8)
C16	0.0741 (14)	0.0539 (12)	0.0439 (11)	−0.0081 (10)	0.0063 (10)	0.0151 (9)
C17	0.0728 (14)	0.0482 (11)	0.0441 (11)	−0.0076 (10)	0.0020 (10)	0.0040 (9)

### Geometric parameters (Å, °)

**Table:**

Ni1—S1	2.1622 (5)	C6—H6A	0.9300
Ni1—S1^i^	2.1622 (5)	C7—C8	1.379 (3)
Ni1—S2^i^	2.1838 (5)	C7—C10	1.501 (3)
Ni1—S2	2.1838 (5)	C8—C9	1.371 (3)
Cl1—C14	1.728 (2)	C8—H8A	0.9300
Cl2—C15	1.7272 (19)	C9—H9A	0.9300
S1—C2	1.737 (2)	C10—H10A	0.9600
S2—C3	1.7388 (19)	C10—H10B	0.9600
N1—C1	1.139 (3)	C10—H10C	0.9600
N2—C4	1.144 (3)	C11—C12	1.465 (2)
N3—C9	1.347 (3)	C11—H11A	0.9300
N3—C5	1.349 (2)	C12—C13	1.380 (3)
N3—N4	1.417 (2)	C12—C17	1.397 (3)
N4—C11	1.260 (3)	C13—C14	1.383 (3)
C1—C2	1.432 (3)	C13—H13A	0.9300
C2—C3	1.359 (3)	C14—C15	1.391 (3)
C3—C4	1.431 (3)	C15—C16	1.378 (3)
C5—C6	1.366 (3)	C16—C17	1.380 (3)
C5—H5A	0.9300	C16—H16A	0.9300
C6—C7	1.383 (3)	C17—H17A	0.9300
			
S1—Ni1—S1^i^	180.0	C7—C8—H8A	119.5
S1—Ni1—S2^i^	88.128 (19)	N3—C9—C8	119.95 (19)
S1^i^—Ni1—S2^i^	91.873 (19)	N3—C9—H9A	120.0
S1—Ni1—S2	91.872 (19)	C8—C9—H9A	120.0
S1^i^—Ni1—S2	88.128 (19)	C7—C10—H10A	109.5
S2^i^—Ni1—S2	180.0	C7—C10—H10B	109.5
C2—S1—Ni1	103.69 (7)	H10A—C10—H10B	109.5
C3—S2—Ni1	103.39 (6)	C7—C10—H10C	109.5
C9—N3—C5	120.77 (17)	H10A—C10—H10C	109.5
C9—N3—N4	113.53 (15)	H10B—C10—H10C	109.5
C5—N3—N4	125.66 (16)	N4—C11—C12	119.38 (18)
C11—N4—N3	117.59 (16)	N4—C11—H11A	120.3
N1—C1—C2	175.0 (3)	C12—C11—H11A	120.3
C3—C2—C1	123.45 (18)	C13—C12—C17	119.61 (18)
C3—C2—S1	120.87 (14)	C13—C12—C11	121.19 (18)
C1—C2—S1	115.62 (15)	C17—C12—C11	119.19 (19)
C2—C3—C4	121.41 (17)	C12—C13—C14	120.30 (18)
C2—C3—S2	120.11 (14)	C12—C13—H13A	119.8
C4—C3—S2	118.47 (14)	C14—C13—H13A	119.8
N2—C4—C3	179.4 (2)	C13—C14—C15	119.98 (19)
N3—C5—C6	119.8 (2)	C13—C14—Cl1	119.36 (16)
N3—C5—H5A	120.1	C15—C14—Cl1	120.64 (15)
C6—C5—H5A	120.1	C16—C15—C14	119.72 (18)
C5—C6—C7	121.3 (2)	C16—C15—Cl2	119.51 (16)
C5—C6—H6A	119.3	C14—C15—Cl2	120.77 (16)
C7—C6—H6A	119.3	C15—C16—C17	120.50 (19)
C8—C7—C6	117.12 (18)	C15—C16—H16A	119.8
C8—C7—C10	120.9 (2)	C17—C16—H16A	119.8
C6—C7—C10	122.0 (2)	C16—C17—C12	119.9 (2)
C9—C8—C7	121.0 (2)	C16—C17—H17A	120.1
C9—C8—H8A	119.5	C12—C17—H17A	120.1

Symmetry codes: (i) −*x*+1, −*y*, −*z*+1.

### Hydrogen-bond geometry (Å, °)

**Table:**

*D*—H···*A*	*D*—H	H···*A*	*D*···*A*	*D*—H···*A*
C5—H5A···N2^ii^	0.93	2.51	3.413 (3)	163

Symmetry codes: (ii) *x*, *y*, *z*−1.
